# A Standardized Precipitation Evapotranspiration Index Reconstruction in the Taihe Mountains Using Tree-Ring Widths for the Last 283 Years

**DOI:** 10.1371/journal.pone.0133605

**Published:** 2015-07-24

**Authors:** Yongyong Ma, Yu Liu, Huiming Song, Junyan Sun, Ying Lei, Yanchao Wang

**Affiliations:** 1 The State Key Laboratory of Loess and Quaternary Geology, Institute of Earth Environment, Chinese Academy of Sciences, Xi’an, 710061, China; 2 The University of the Chinese Academy of Science, Beijing, 100049, China; 3 Joint Center for Global Change Studies (JCGCS), Beijing Normal University, Beijing, 100875, China; 4 Department of Geography, Xingtai University, Xingtai, 054001, China; Institute of Botany, CHINA

## Abstract

Tree-ring samples from Chinese Pine (*Pinus tabulaeformis* Carr.) that were collected in the Taihe Mountains on the western Loess Plateau, China, were used to analyze the effects of climate and drought on radial growth and to reconstruct the mean April-June Standardized Precipitation Evapotranspiration Index (SPEI) during the period 1730–2012 AD. Precipitation positively affected tree growth primarily during wet seasons, while temperature negatively affected tree growth during dry seasons. Tree growth responded positively to SPEI at long time scales most likely because the trees were able to withstand water deficits but lacked a rapid response to drought. The 10-month scale SPEI was chosen for further drought reconstruction. A calibration model for the period 1951–2011 explained 51% of the variance in the modeled SPEI data. Our SPEI reconstruction revealed long-term patterns of drought variability and captured some significant drought events, including the severe drought of 1928–1930 and the clear drying trend since the 1950s which were widespread across northern China. The reconstruction was also consistent with two other reconstructions on the western Loess Plateau at both interannual and decadal scales. The reconstructed SPEI series showed synchronous variations with the drought/wetness indices and spatial correlation analyses indicated that this reconstruction could be representative of large-scale SPEI variability in northern China. Period analysis discovered 128-year, 25-year, 2.62-year, 2.36-year, and 2.04-year cycles in this reconstruction. The time-dependency of the growth response to drought should be considered in further studies of the community dynamics. The SPEI reconstruction improves the sparse network of long-term climate records for an enhanced understanding of climatic variability on the western Loess Plateau, China.

## Introduction

During recent decades, drought events have increased in intensity and frequency, particularly in arid and semi-arid regions, in response to global climate change [1, 2]. This drought variation has strongly affected regional social and agricultural developments, resulting in significant economic losses in northern China. Therefore, investigating the sensitivity of vegetation to drought, the characteristics of drought variation and the potential forcing mechanisms of drought is essential and can improve our knowledge regarding vegetation vulnerability to climate change and our ability to predict future drought variations.

Limited by time span and space coverage, direct instrumental records can only offer limited insights into current drought variations. Thus, the long-term drought variation history must be recovered using proxy records. With their high resolution and accurate dating method, tree rings are universally acknowledged as one of the most valuable proxies and have played a crucial role in paleoclimate research and ecological investigations [3]. In the past decades, there have several dendroclimatological studies on the western Loess Plateau, such as temperature [4, 5], precipitation [6, 7] and drought [8–10] reconstructions. However, tree ring data are still not sufficient.

The Standardized Precipitation Evapotranspiration Index (SPEI) is a multiscalar drought index based on climatic data, which allows this index to be used to detect, monitor, and analyze drought events [11]. Based on precipitation and potential evapotranspiration, the SPEI combines the sensitivity of the Palmer Drought Severity Index (PDSI) to changes in evaporation demand with the simple calculations and the multitemporal nature of the Standardized Precipitation Index (SPI) [12]. The SPEI has been widely used to analyze and evaluate drought events worldwide [13–16]. Combining tree rings and the SPEI together for the purpose of studying the community dynamics of plants affected by drought has been achieved in some areas [17–20]; however, this method has been used only rarely in China.

The goals of this study were as follows: (1) to determine the primary climatic variables related to radial tree growth, (2) to evaluate the effects of climate and drought on tree growth at different time scales, (3) to combine tree-ring width and the SPEI to reconstruct a seasonal drought history over the past 300 years using tree-ring widths from the Taihe Mountains, and (4) to investigate decadal to multi-decadal scale drought variations on the western Loess Plateau.

## Materials and Methods

### Study area and chronology development

**Fig 1:**
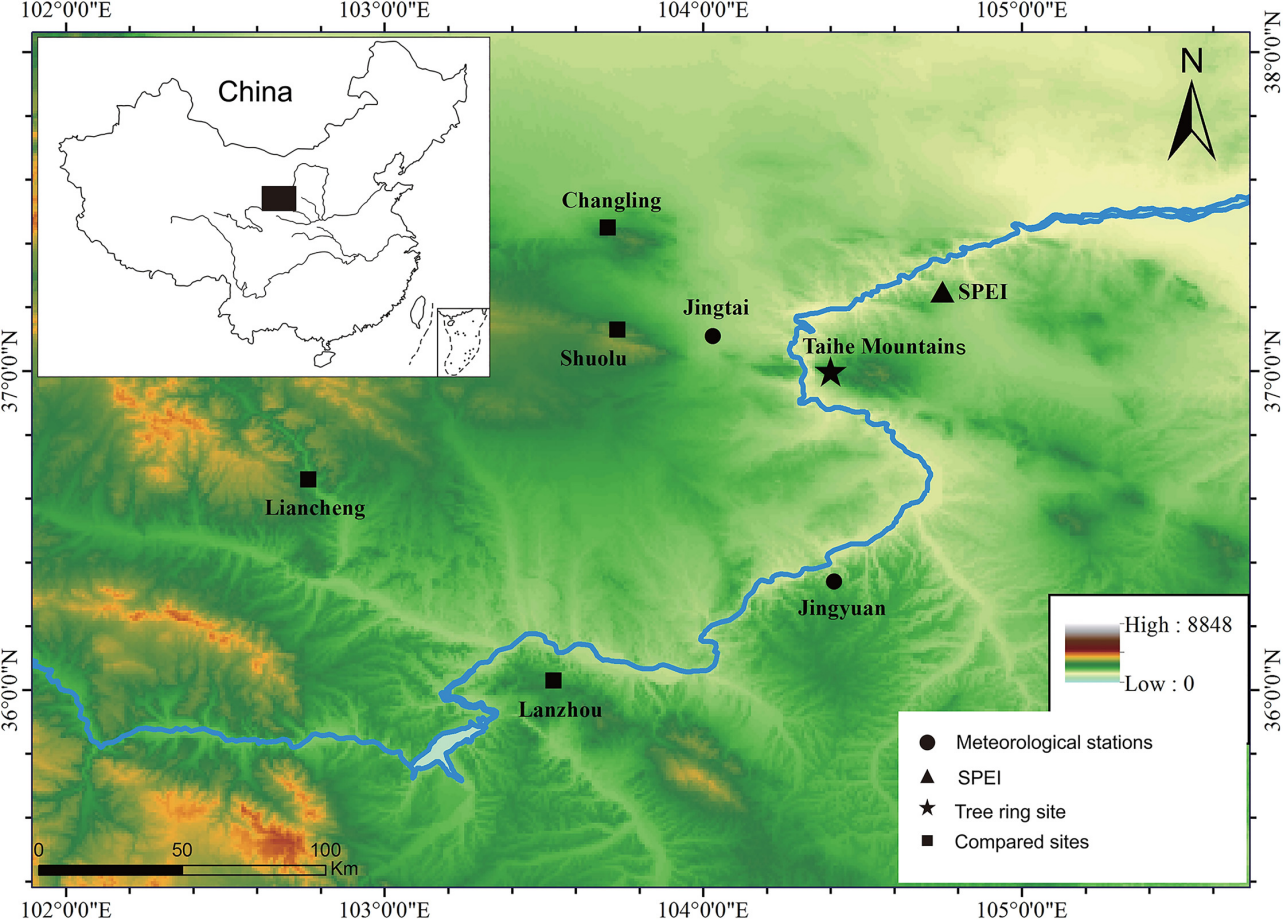
Map of the sampling site.

Our sampling site (104°36′E, 37°N; elevation 2400–2700 m) was in the Taihe Mountains in the mid-eastern Gansu Province and in the upper reaches of the Yellow River (Fig 1). The Taihe Mountains, which is composed of metamorphic rock, granite, and diorite, primarily formed by an uplift process on the north side of the eastern Qilian Mountains. The Taihe Mountains has a temperate continental monsoonal climate with four distinct seasons. The microclimate, which is influenced by the forest environment, is relatively cold and wet in the forest. The annual mean temperature is 6–7°C, and the annual precipitation is 350–410 mm, with the majority of precipitation falling from July to September [21]. Chinese pines (*Pinus tabulaeformis*), which are the dominant tree species, are generally distributed vertically on the steep nightside at an elevation range of 2200–2700 m. No specific permissions were required for this sampling site, and the field studies did not involve endangered or protected species.

One to two cores were collected from each tree. In total, 52 cores from 27 Chinese pines were collected from the Taihe Mountains. The samples were dried, surfaced, and cross-dated before measured at a resolution of 0.01 mm according to standard dendrochronological procedures [22]. The cross-dating quality control procedure was performed using the COFFECHA program [23]. The master series covered the period from 1692 to 2012, and the mean length of the entire series was 250.6 years. The intercorrelation of all raw measuring series was 0.785, and the average mean sensitivity was 0.501, demonstrating the reliability of the cross-dating and a common variation pattern among these tree-ring series.

**Fig 2:**
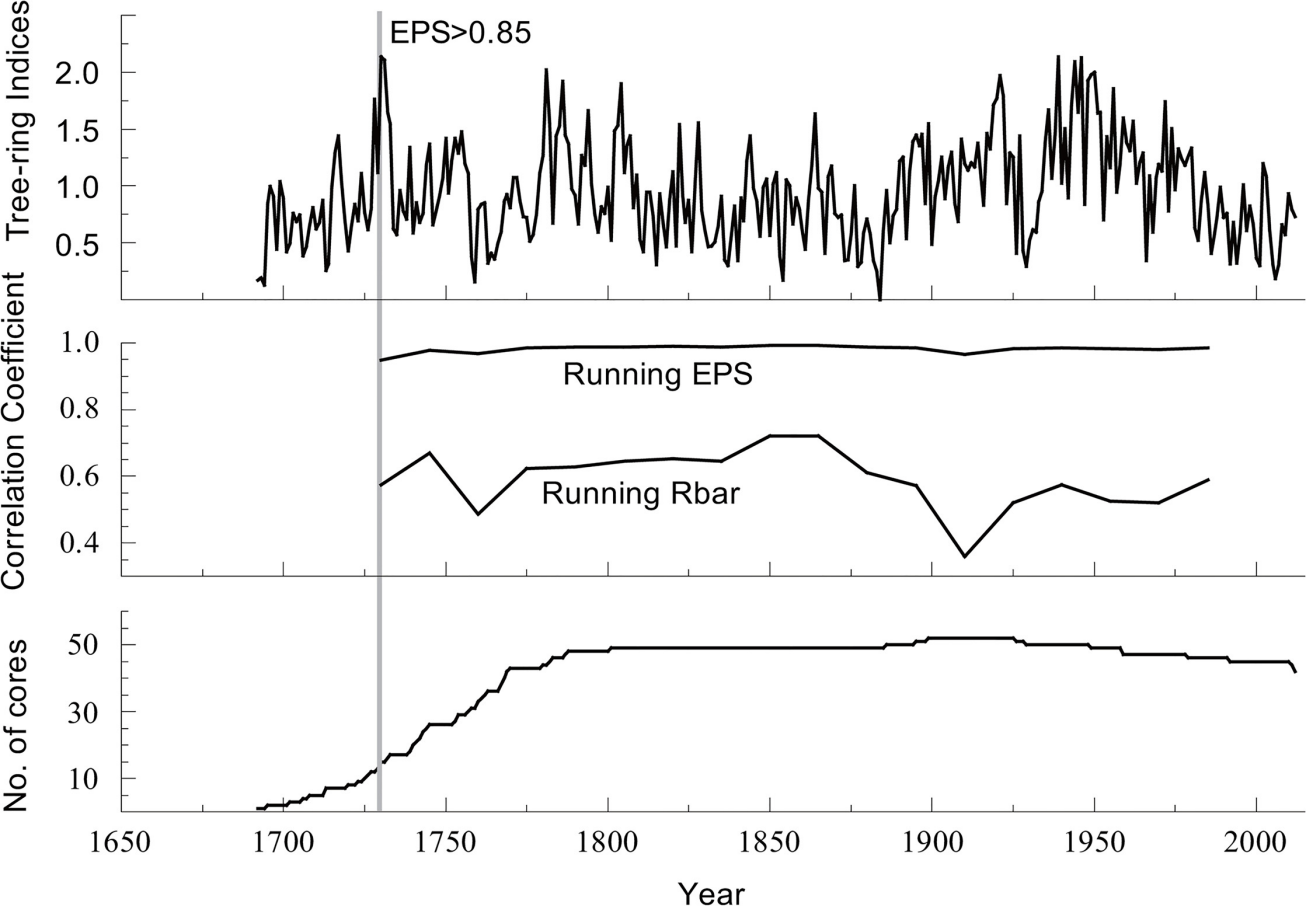
Tree-ring STD chronology, running EPS, running Rbar and sample size.

The chronology was developed using the ARSTAN program [24]. We used a conservative method that used negative exponential curves or straight lines of any slope to fit each ring width measurement series in order to remove the undesirable growth trends related to age and stand dynamics, which are unrelated to climatic variations, and to preserve the maximum common signals at the lowest frequency as far as possible [24]. Then, the individual index series were combined into a single chronology by calculating a bi-weight robust mean [24]. Finally, we obtained three types of chronologies: standard (STD) chronology, residual (RES) chronology, and ARSTAN (ARS) chronology. The STD chronology was used in the further analysis because this chronology preserved a greater number of low frequency signals [24]. The expressed population signal (EPS), which indicates the degree to which a particular sample chronology portrays a hypothetically perfect chronology [25], was computed using a 30-year moving window with 15-year overlaps to assess the reliability of the chronology over time. An EPS threshold value of 0.85, which corresponded to a minimum sample depth of ten cores from 1730 AD, was employed in this paper (Fig 2).

### Climatic and SPEI data

**Fig 3:**
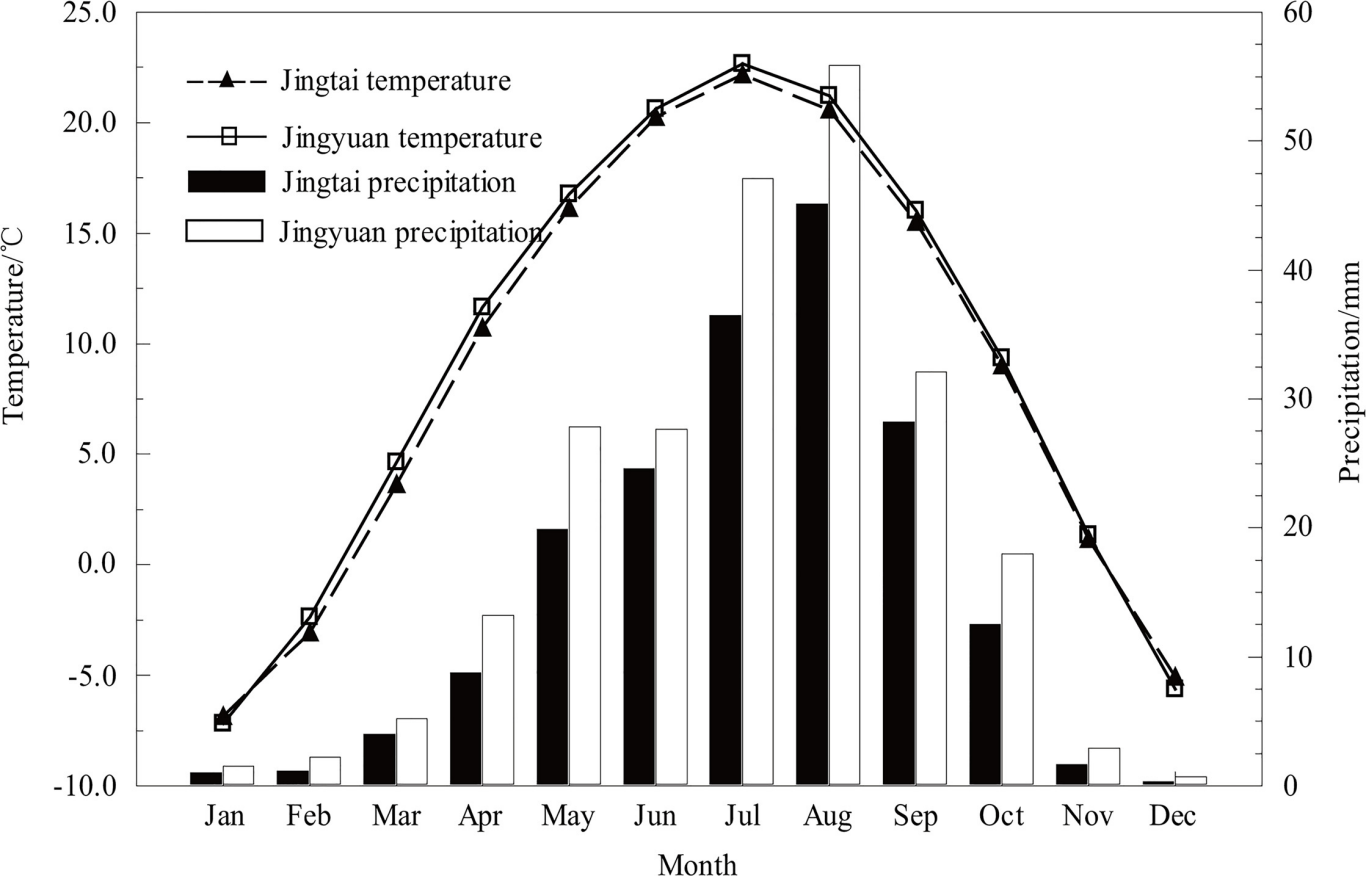
Monthly mean temperature and total precipitation values at Jingtai (1957–2013) and Jingyuan (1951–2013) meteorological stations.

The local monthly temperature and precipitation records from the two closest meteorological stations, Jingtai station (37.11°N, 104.03°E, elevation 1630.9 m, 1957–2013 AD) and Jingyuan station (36.34°N, 104.41°E, elevation 1398.2 m, 1951–2013 AD), were used in this paper (Fig 1). The temperature and precipitation data obtained from these two stations were tested for homogeneity and randomness using the double-mass method [26] and the Mann-Kendall method [27]. The results of these tests indicated that these data qualified for further analysis. As shown in Fig 3, the mean monthly precipitation and temperature values of these two stations have synchronous variations. High precipitation is concentrated from July to September, and the highest temperature is in July. The monthly precipitation at Jingyuan station (average annual total precipitation is 234 mm) is marginally higher than that at Jingtai station (average annual total precipitation is 184 mm). Regardless, we used the average values of the meteorological data at the two sites to reflect the regional mean climate condition during the instrumental period.

The drought index used in this article is the SPEI. The SPEI values are always range from -5–5. The smaller values indicate stronger degrees of drought, and larger values indicate higher degrees of moisture. The use of the SPEI is relevant for quantifying the effects of droughts on tree growth at different time scales [19]. In the present study, we adopted the global gridded SPEI dataset with a 0.5° spatial resolution at a time scale between 1 and 24 months [28] (http://sac.csic.es/spei/index.html). The monthly SPEI data during the period from 1951 to 2011 in the nearest gridded dataset (37.25°N, 104.75°E, Fig 1) were eventually used in our further drought analyses.

### Statistical methods

Climate-growth relationships were analyzed by using correlation functions [29] between the STD chronology and the climate data. The precipitation, temperature, and SPEI data records from July of the previous year through October of the current year were selected to identify the climatic effects on the radial growth of Chinese pine in the study region. Then, a simple linear regression model was employed to reconstruct the historical series [24] and a split calibration-verification procedure was used to validate the reconstruction [30]. The statistics provided for the calibration period were the Pearson’s correlation coefficient(*r*) and the coefficient of determination (*R* ^2^), and the fidelity of the calibrations during the verification period were assessed via Pearson’s correlation coefficient (*r*), the explained variance (*r* ^2^), the sign test, the reduction of error (RE), and the coefficient of efficiency (CE). In general, the RE and CE are the most rigorous statistical analyses, and CE or RE values >0 indicate good model fit [31].

## Results and Discussion

### Climate response

**Fig 4:**
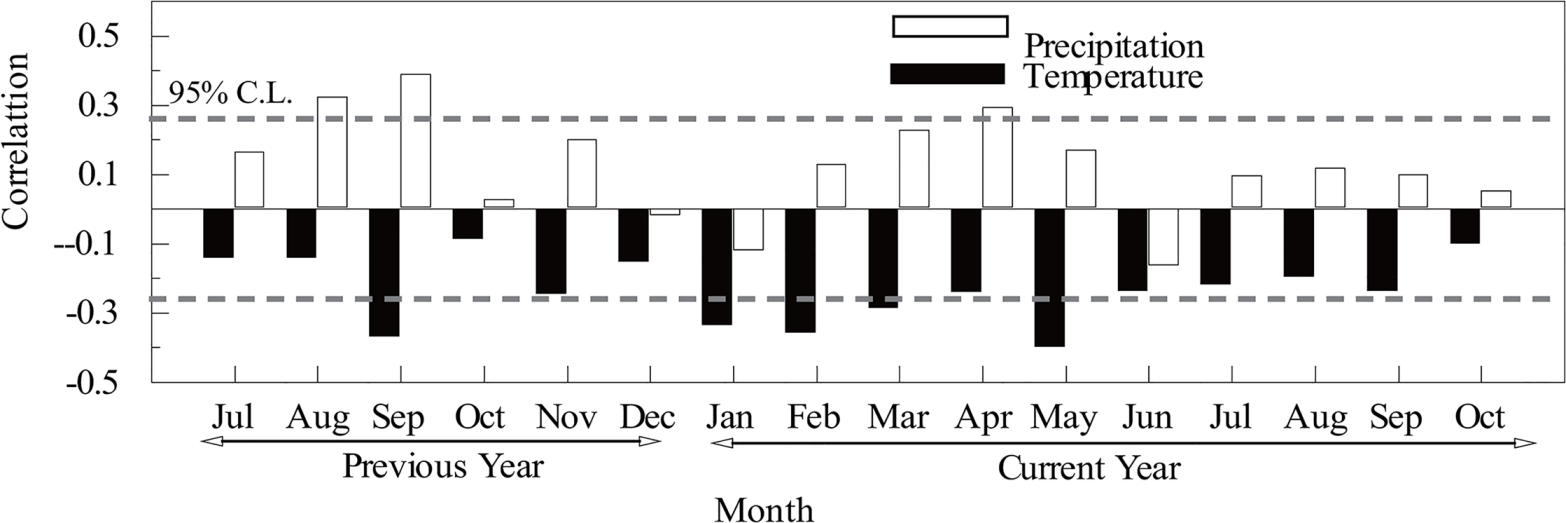
Correlations between the tree-ring STD chronology and monthly mean temperature, and monthly total precipitation (1957–2012 AD). Horizontal dashed lines represent the 95% C.L.

As shown in Fig 4, significantly positive correlations (at the 95% confidence level (C.L.)) were found between the STD chronology and precipitation during August and September of the previous year and during April of the current year. Significantly negative correlations were found between the STD chronology and temperature during September of the previous year and during January, February, March and May of the current year. The strongest growth responses to precipitation were observed for those months included primarily within the wet season in summer, while the strongest growth responses to temperature occurred primarily in winter and spring when high temperature would enhance evaporation and further reduce available water at the beginning of the growing season. After seasonal combinations, the strongest response of growth indices to precipitation was found from August of the previous year and May of the current year (*r* = 0.566). Additionally, the strongest response of growth indices to temperature was found from January of the current year to May of the current year (*r* = -0.524), suggesting that both precipitation and temperature strongly correlated with tree growth. In general, the overall climate-growth response negatively correlates with temperature and positively correlates with precipitation, revealing the moisture-stressed growth pattern in the study region [3]. This moisture-stressed growth pattern is also found in other dendroclimatic studies on the Loess Plateau [9, 10, 32].

**Fig 5:**
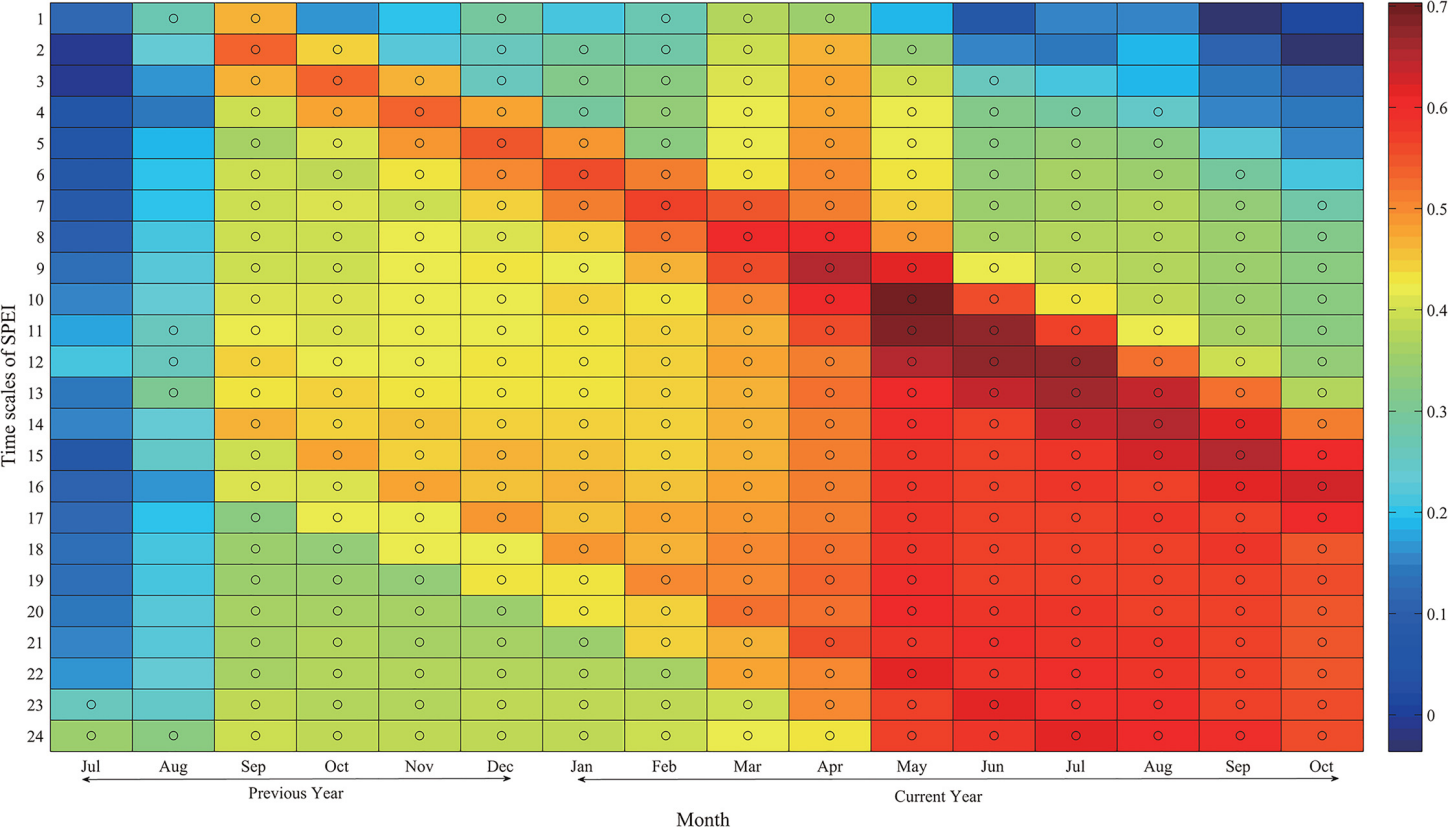
Correlations between the STD chronology and monthly SPEI data at time scales from 1 to 24 months (AD 1951–2011). Circles represent significant at the 95% C.L.

As shown in Fig 5, the tree-ring chronology showed positive significant correlations with the SPEI data irrespective of the analyzed time scale, indicating that tree growth is severely limited by water deficits. Most significant correlations occurred at time scales between 10 and 13 months, indicating that the Chinese pines in the study area can’t be affected significantly by drought unless they are exposed to sustained water deficits, namely those water deficits registered by the long timescales of the SPEI [19]. The reason may be that the Chinese pines in the study region have adapted to water shortage and have physiological mechanisms allowing them to cope with these conditions [33]. This finding is consistent with a global study which evidenced that vegetation activity and growth in semiarid biomes primarily responded to drought at long time scales [19].

Based on the above analysis, we chose the SPEI at the 10-month scale for the further drought analysis and reconstruction. All of the correlations are significant at the 95% C.L. except for the correlation with July and August of the previous year (Fig 5). This significant correlation between tree-ring chronology and the SPEI data are probably due to that the study site is located in a very dry area. After seasonal combinations, the highest correlation between the tree-ring chronology and the seasonal SPEI occurred during the period from April to June (*r* = 0.716; 1951–2011; *p*<0.0001). This seasonal pattern is quite similar to the findings reported in previous studies [10, 34].

### Transfer function and SPEI reconstruction

The mean SPEI from April to June (SPEI_AJ_) for the last 283 years was reconstructed based on the above analyses. The results of the split-sample calibration-verification tests are shown in Table 1. Except for the sign text, all the statistics are significant at 99% confidence. The values of the two most rigorous tests of model validation, the RE and CE, were both positive, indicating the significant fit of the regression model. During the full calibration period 1951–2011, the reconstruction accounted for 51% of the actual SPEI_AJ_ variance. These results demonstrated the validity of our regression model.

**Table 1:** Statistics for calibration and verification test results.

Calibration			Verification					
period	*r*	*R* ^2^	Period	*r*	*r* ^2^	RE	CE	Sign test
1951–1981	0.72^a^	0.52	1982–2011	0.63^a^	0.40	0.55	0.27	30/12^b^
1981–2011	0.63^a^	0.40	1951–1980	0.69^a^	0.48	0.58	0.24	30/11^b^
1951–2011	0.72^a^	0.51	-	-	-	-	-	-

^a^ Significance at the 99% confidence level

^b^ Significance not at the 95% confidence level

**Fig 6:**
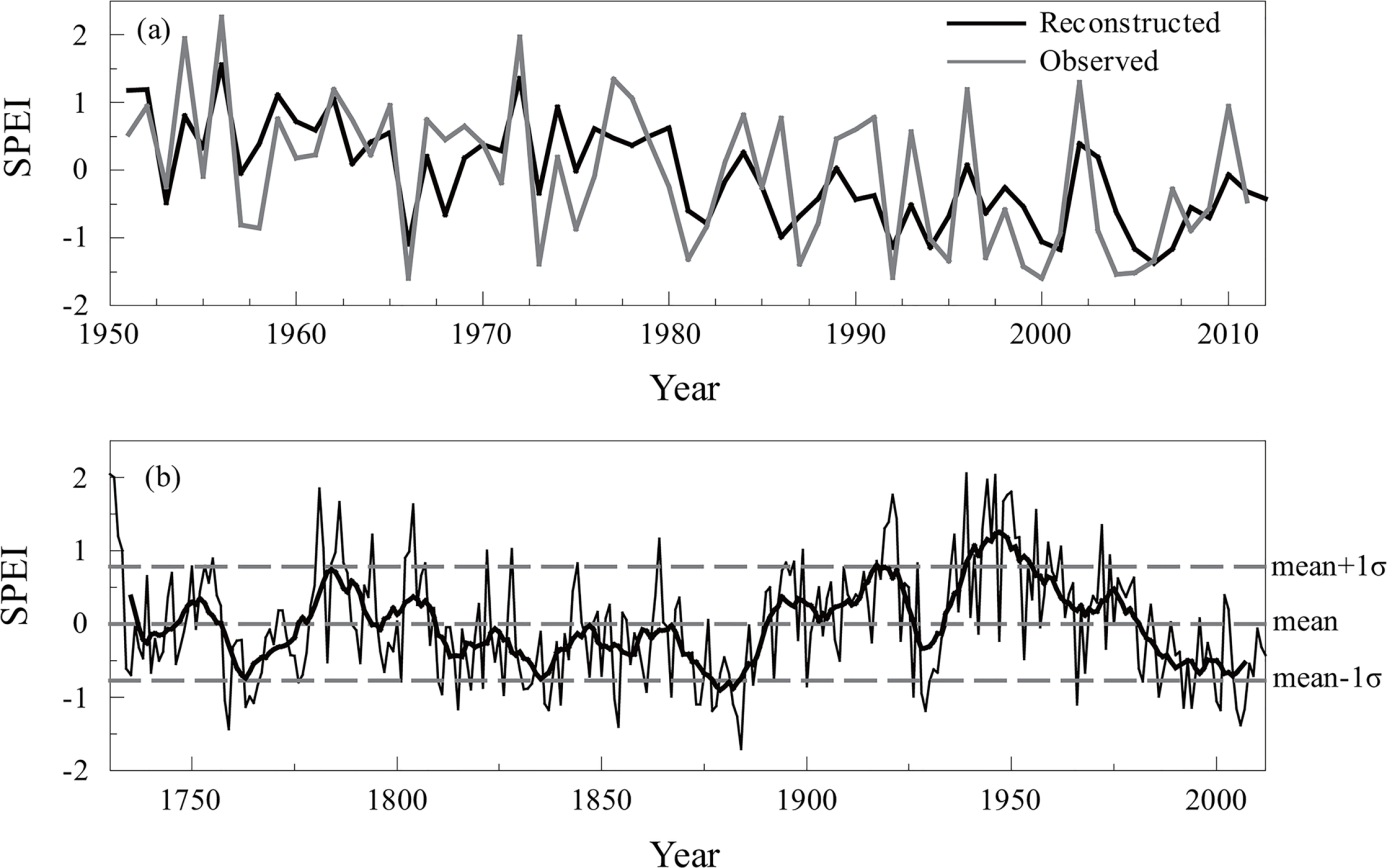
Comparison between observed and reconstructed SPEI_AJ_. (a) The reconstructed (black line) and observed (gray line) SPEI_AJ_ during 1951–2011. (b)The reconstructed SPEI_AJ_ after an 11-year moving average for the Taihe Mountains from 1730 to 2012.

Then, a transfer function between tree-ring width and the SPEI_AJ_ was designed as follows:

$${\text{SPEI}}_{\text{AJ}}=1.756\times {\text{W}}_{t}-1.704$$ (1)

where SPEI_MA_ is the mean SPEI from April to June and W_t_ is the tree-ring index at year t. As shown in Fig 6A, the reconstructed SPEI_MA_ visually tracked the observations quite well. Notably, two series remained significantly correlated after applying linear detrending (*r* = 0.64, *p*<0.01) or first differenced transformation (*r* = 0.77, *p*<0.01) to both the observation and the reconstruction, indicating that the reconstruction also captured the real climatic signal at high frequency.

### Drought on the Taihe Mountains

The mean value of our SPEI_AJ_ reconstruction since 1730 AD was 0.002, and the standard deviation σ was 0.778. We defined wet years as those years that were greater than mean+1σ, and drought years as those years that were less than mean−1σ (Fig 6B, see data in S1 Text). Then, during the last 283 years, 50 years were categorized as wet years (accounting for 17.67% of the total), and 46 years were categorized as drought years (accounting for 16.25% of the total), with the remaining 187 years categorized as normal years. In general, the long-lasting drought or wet events have much stronger effects on local social and economic activities. Then, we analyzed the multi-year continuous droughts for the last 283 years based on the drought history reconstruction. Droughts lasting over three years were found in 1763–1766, 1831–1833, 1882–1884, and 1928–1930, among which the drought epoch of 1882–1884 was the most severe drought in the reconstruction since 1730 AD. Another drought epoch in 1928–1930, which occurred over a large geographic area in Northern China and which has been documented by many studies of the surrounding regions [35–42], was also captured in our drought reconstruction. Meanwhile, long-lasting wet spells were found in 1730–1733, 1784–1787, 1702–1704, 1919–1922, 1943–1946, and 1948–1952. The wet epoch of 1943–1952 was the longest wet period, with the exception of a sharp decrease in the SPEI_AJ_ value in 1947. This long-lasting wet epoch was also documented in other dendroclimatic studies [6, 9].

After smoothing using an 11-year moving average, many fluctuations emerged at the decadal scale. The low frequency wet periods with SPEI_AJ_ values above the long-term mean (0.002) occurred in 1730–1736, 1747–1757, 1776–1793, 1797–1809, 1891–1926, and 1934–1981. And the dry periods occurred in 1737–1746, 1758–1775, 1794–1796, 1810–1890, and 1927–1933. The twentieth century had the most noteworthy feature in our reconstruction. Following the drought in the late 1920s, the wettest period of the past 283 years occurred in the 1940s, and a drying trend has persisted since the 1950s, which is consistent with the assessment based on the available meteorological records in central, northern, and northeastern China [43, 44].

### Comparison with dryness/wetness indices

**Fig 7:**
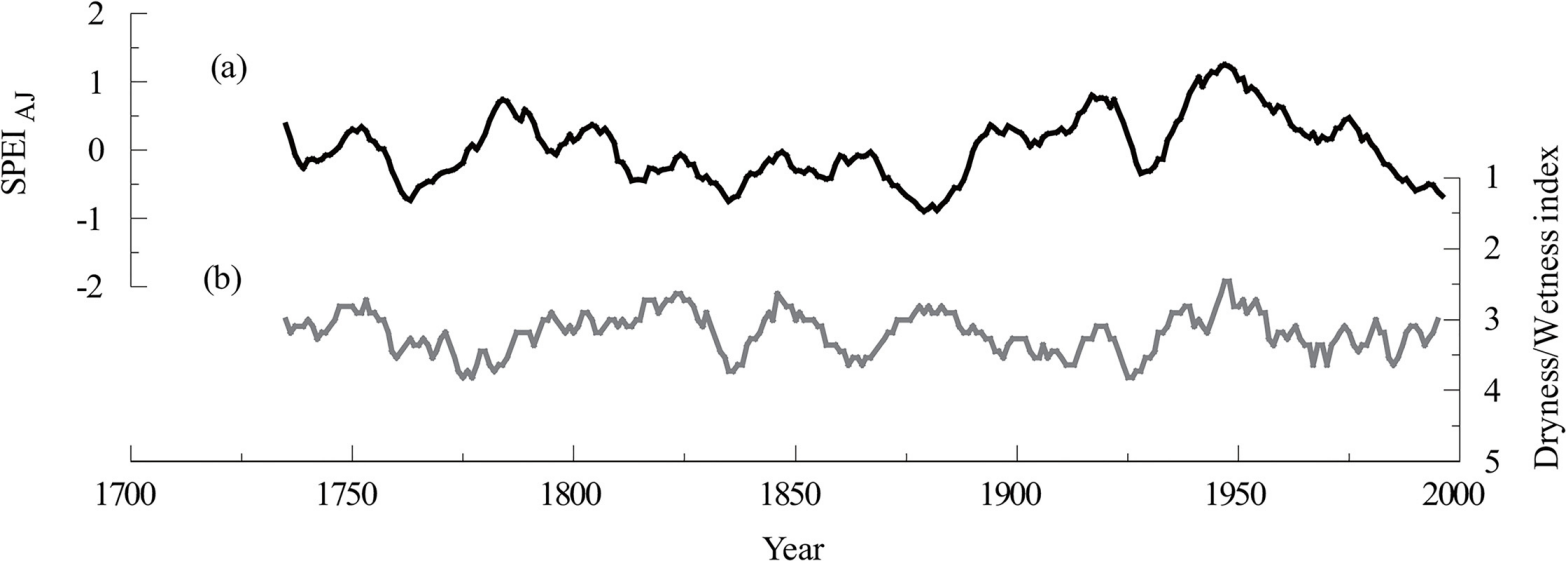
Comparison between the SPEI_AJ_ reconstruction and dryness/wetness indices of Lanzhou.

Dryness/wetness indices derived from historical documents [45] are of great importance in studying historical climate changes, which can be compared and crosschecked with tree-ring-based climate reconstructions. The dryness/wetness indices are classified into five grades: wettest, wet, normal, dry and drought, which corresponded to values ranging from 1 to 5 [45]. In this paper, the reconstructed drought series was compared with the dryness/wetness indices of Lanzhou which is near the study area. The correlation coefficient between the reconstruction and the dryness/wetness indices was -0.17 (n = 271, 1730–2000, *p*<0.01) and these indices showed some similar trends at the decadal scale (Fig 7). However, some notable differences were observed between the dryness/wetness series and our reconstruction. For example, during the periods of 1770–1790 and 1860–1890, the reconstruction and the dryness/wetness series displayed inverse dry/wet phases. The discrepancies between tree-ring records and dryness/wetness data may be attributed to omissions and inconsistencies in historical records and to misrepresentations of historical facts [46], which have also been found in other studies [41, 47, 48].

### Comparison with other tree-ring-based reconstructions

**Fig 8:**
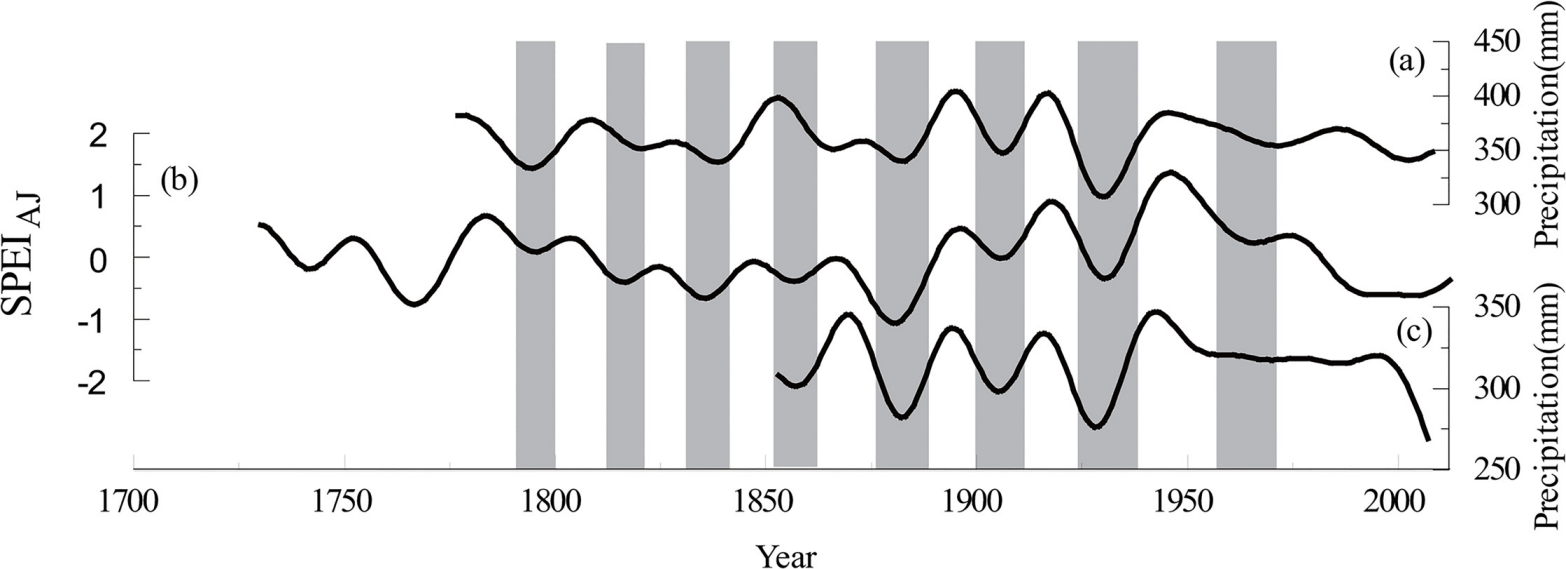
Tree-ring-based reconstruction comparisons among three sites. (a) The reconstructed precipitation in Liancheng. (b) The SPEI_AJ_ reconstruction in the Taihe Mountains. (c) The reconstructed precipitation in the Changling-Shoulu region.

The reconstructed SPEI_AJ_ series were also compared with the reconstructed precipitation from the Changling-Shoulu region [6] and Liancheng [7]. The three curves were significantly correlated at inter-annual and decadal scales. The correlation between the SPEI_AJ_ reconstruction and the precipitation reconstruction of Changling-Shoulu region was 0.60 (n = 155, 1853–2007, *p*<0.0001), and the correlation between the SPEI_AJ_ reconstruction and the precipitation reconstruction of Liancheng was 0.51 (n = 232, 1777–2008, *p*<0.0001). Additionally, the wet and dry periods of the three curves agreed well with each other on the decadal scale, particularly during the dry periods (Fig 8), reflecting that precipitation may be a major contributor to drought in the study area.

### Spatial representativeness

**Fig 9:**
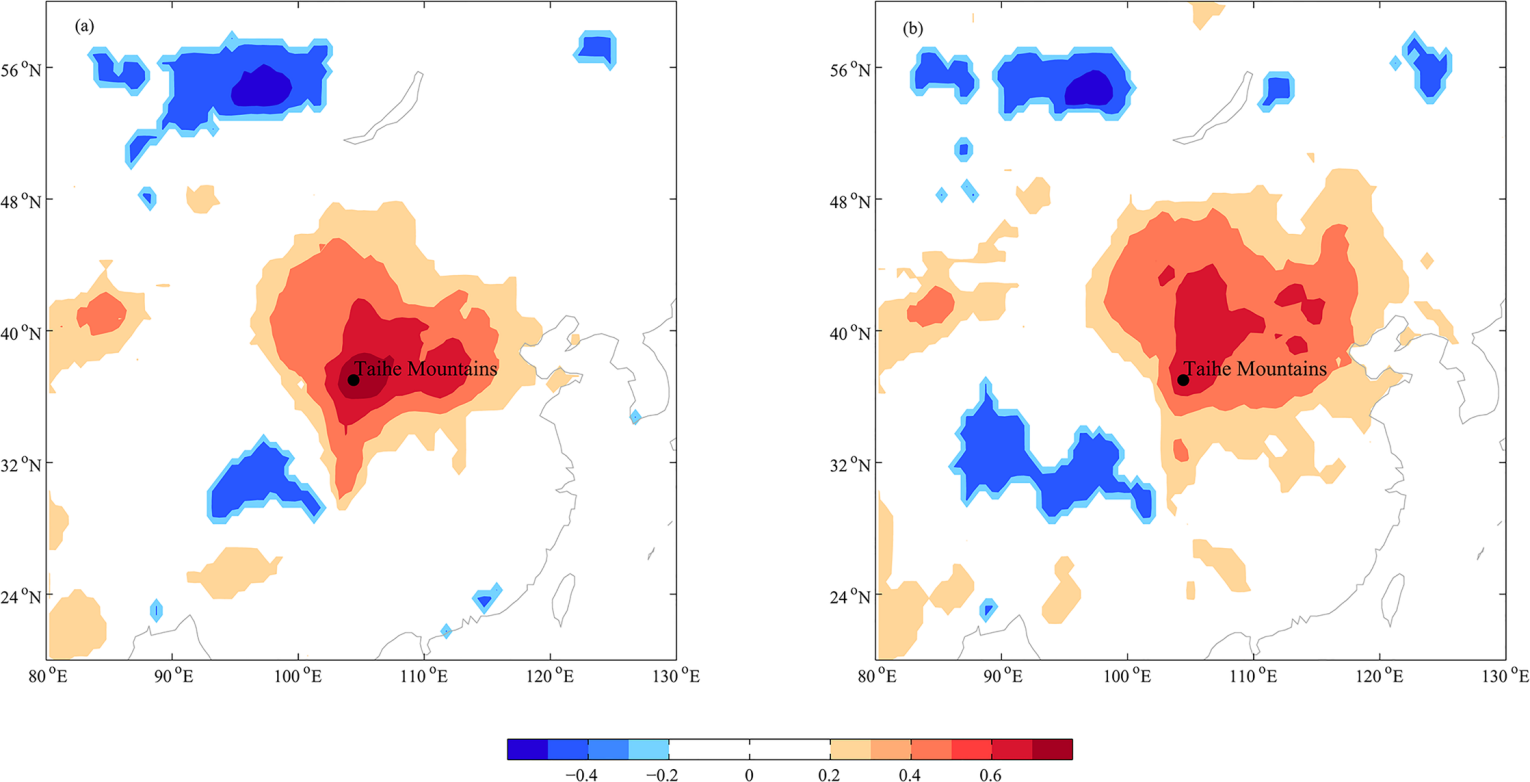
Patterns of field correlation in our study. (a) Correlations between the gridded (37.25°N, 104.75°E) SPEI_AJ_ series with the 0.5×0.5 gridded mean April-June SPEI at the 10-month scale. (b) Correlations between reconstructed SPEI_AJ_ with the 0.5×0.5 gridded mean April-June SPEI at the 10-month scale (1951–2011 AD).

To understand the regional representativeness of our SPEI_AJ_ reconstruction, spatial correlation was performed between the reconstructed and instrumental SPEI_AJ_ series with the global SPEI_MA_ datasets (Fig 9). The spatial correlation fields were similar for the instrumental and reconstructed SPEI_AJ_. The SPEI_AJ_ reconstruction in the study region had a significant positive correlation over a large region in northern China. Therefore, the SPEI_AJ_ reconstruction at the Taihe Mountains is representative of the SPEI_AJ_ variations for a large region.

### Periodicities in the Taihe SPEI_AJ_ reconstruction

**Fig 10:**
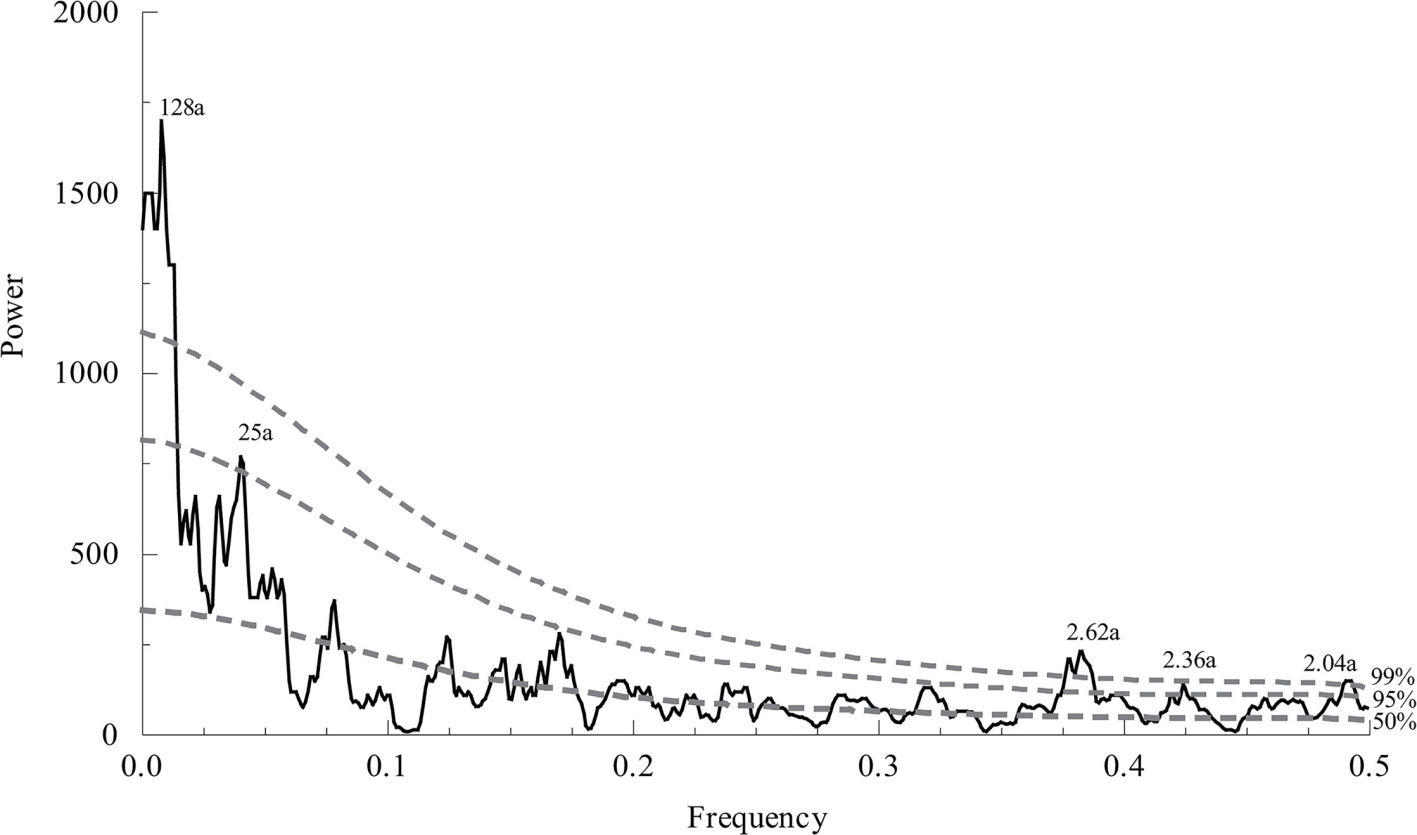
MTM spectral density of the reconstruction. The 50% line indicates the null hypothesis, and the 95% and 99% lines indicate the relevant confident levels. Some periodicities passing 95% are labeled in the plot

The multi-taper method (MTM) of spectral analysis [49] was further performed on the SPEI_AJ_ reconstruction (1730–2012 AD) to detect the periodicities of drought history (Fig 10). The 128-year and 25-year cycles may suggest the influences of solar effects. The 128-year cycle should be treated cautiously regarding the length of the reconstruction. Significant high-frequent peaks were found at 2.62-year (99% C.L.), 2.36-year (95% C.L.) and 2.04-year (99% C.L.) interannual cycles. All these interannual cycles fall within the range of El Niño-Southern Oscillation (ENSO) variability [50], and the 2.04–year cycle also resembles the variability of tropical biennial oscillation [51]. This finding is consistent with early studies based on the available meteorological records in northern China [52, 53], suggesting that large-scale ocean–atmosphere–land circulation systems may have strong effects on the drought variations in the study area.

## Conclusions

A tree-ring width chronology from 1692 to 2012 in the Taihe Mountains, Gansu Province, China was developed in this study. This chronology is most reliable after 1730 for the period in which the EPS is greater than 0.85. Climate response analysis indicated that Chinese pines in the study area were moisture-stressed and responded to drought at long time scales. As indicated in our study, regional drought variability has increased during the twentieth century, and a clear drying trend has occurred since the 1950s. The reconstruction showed good spatial agreement with the global gridded SPEI_AJ_ dataset and similar dry–wet fluctuations to the reconstructed precipitation from the Changling-Shoulu region and Liancheng. Spectral analysis detected significant cycles that were possibly related to ENSO activity, the tropical biennial oscillation, and solar influences, suggesting that the drought variations in the study area may be influenced by multiple large-scale climate forcings. The results of this study proved the feasibility of combining tree rings and the SPEI for studying the sensitivity of vegetation to drought and drought reconstructions on the western Loess Plateau, China. However, future studies that develop longer tree-ring chronologies and more reconstructions with additional species and with larger spatial coverage over the surrounding areas are of critical importance to better understand the effects of droughts on different ecological systems and the forcing mechanisms of the droughts.

## Supporting Information

S1 Text SPEI reconstruction in the Taihe Mountains, China. The data includes the reconstructed April-June SPEI series.
(TXT)
